# Effects of Intensive Control of Glycemia on Clinical Kidney Outcomes in Type 2 Diabetes Patients Compared with Standard Control: A Meta-Analysis

**DOI:** 10.3389/fphar.2017.00845

**Published:** 2017-11-21

**Authors:** Francisco Herrera-Gómez, María Asensio-González, Anunciación González-López, F. Javier Álvarez

**Affiliations:** ^1^Pharmacology and Therapeutics, Faculty of Medicine, University of Valladolid, Valladolid, Spain; ^2^Nephrology, Complejo Asistencial de Zamora, Zamora, Spain; ^3^CEIC/CEIm Área de Salud Valladolid Este, Hospital Clínico Universitario de Valladolid, Valladolid, Spain

**Keywords:** blood glucose, diabetes mellitus, type 2, Hemoglobin A, glycosylated, humans, kidney

## Abstract

**Background:** Association between poor control of glycemia and the onset of microvascular complications in type 2 diabetes mellitus (T2DM) patients is a hard issue. However, it seems that the impact of pharmacological treatment is important only in early stages of diabetic nephropathy. We sought to examine whether intensive glycemic control is associated with improvement of clinical Chronic Kidney Disease (CKD) outcomes compared to standard glycemic control.

**Methods:** Meta-analysis of published and unpublished randomized controlled trials (RCT) and *post-hoc* analysis of RCTs comparing anti-diabetic drugs and/or insulin (intensive control) vs. dietary measures (standard control) for relevant outcomes related to progression of CKD clinically manifest was undertaken. Summary estimates obtained by random effects model and funnel plots for assessing reporting bias are presented.

**Results:** Our analysis was based on four RCTs representing 27,391 adult T2DM patients with CKD from around the world. The pooled OR for the outcomes of doubling of serum creatinine and need of dialysis were, respectively, of 0.98 with 95% confidence interval (95% CI) 0.81–1.19, and 0.84 with 95% CI 0.69–1.02. The pooled OR for the outcome of death from kidney failure was 0.62 with 95% CI 0.39–0.98. Clinical differences between studies were not translated in statistical heterogeneity. Reporting bias may be present.

**Conclusions:** Intensive glycemic control has an effect on death from kidney failure compared to standard glycemic control. Better comprehension of glycemic control effects on both T2DM patients with and without CKD is important for individualization of these two treatment modalities.

## Introduction

Type 2 diabetes mellitus (T2DM) is a common disease that is increasing rapidly in prevalence worldwide (Shi and Hu, 2014; IDF Diabetes Atlas, 2017). Association between high blood glucose concentrations and microvascular complications is evident from the results of several studies showing that glycemic control can delay the onset and progression of diabetic retinopathy, nephropathy, and neuropathy (Klein et al., 1984; Ballard et al., 1988; Diabetes Control and Complications Trial Research Group, 1993; Ohkubo et al., 1995; Adler et al., 1997). It has been observed that urinary albumin excretion is prevented using insulin, and particularly with multiple injections (Diabetes Control and Complications Trial Research Group, 1993; Ohkubo et al., 1995). However, it seems that the impact of pharmacological treatment is present only in early stages of diabetic nephropathy, and little is known about the influence of glucose-lowering medications on clinical kidney disease outcomes. Although experts and guidelines continue to recommend rigorous glycated hemoglobin (HbA_1c_) targets (National Kidney Foundation, 2012; Handelsman et al., 2015; Inzucchi et al., 2015), achieving glycemic thresholds does not benefit patients with advanced CKD (1998a; Adler et al., 2003; ADVANCE Collaborative Group, 2008; Holman et al., 2008; Duckworth et al., 2009; Ismail-Beigi et al., 2010; Perkovic et al., 2013; Papademetriou et al., 2015; Mohammedi et al., 2016).

It is important to note that more intensive glycemic control is associated with hypoglycemia as kidney function decreases (CKD stages 3–5) (Jun et al., 2011), basically due to an effective increase in the duration of action of insulin as a consequence of decreased clearance of it (Alsahli and Gerich, 2014). Hypoglycemia, as a marker of poorer clinical outcomes, has been addressed by studies showing increased mortality in patients under intensive glycemic therapy (Action to Control Cardiovascular Risk in Diabetes Study Group, 2008; ADVANCE Collaborative Group, 2008; Duckworth et al., 2009). Appetite decreases as CKD progresses, leading to a reduced intake, which is at the same time responsible of hypoglycemic events (Walker et al., 2014). Reducing intake is also responsible of malnutrition that is considered one of the most important risk factors for hypoglycemia (Garla et al., 2017). An abnormal catabolic state maintained by inflammation and biochemical changes inherent to CKD (not controlled adequately if patients do not adhere to medications) contribute finally to perpetuate malnutrition and favor more frequent hypoglycemic events (Park et al., 2012; Garla et al., 2017).

Accordingly, attempts to tighten glycemic control are well-considered by clinicians, but only if there is no risk for hypoglycemia. Nevertheless, dietary restrictions may be not sufficient compared to pharmacologic measures, even in advanced CKD patients who are not able to tolerate any dose of insulin (Rhee et al., 2014). The rapid passage from hyperglycemia to hypoglycemia is frequently observed in these patients in whom an optimal glycemic control is particularly challenging.

Faced with such difficulties and taken into account the conflicting results from randomized controlled trials (RCT), in the context of current strategies, this meta-analysis sought to examine whether intensive glycemic control is associated, or not, with improvement of clinical CKD outcomes compared to standard glycemic control.

## Materials and methods

This meta-analysis was conducted and reported in compliance with the Preferred Reporting Items for Systematic Reviews and Meta-Analyses (PRISMA) guidelines (Shamseer et al., 2015). A systematic review protocol was developed, refined, and registered in the International Prospective Register of Systematic Reviews (PROSPERO) on 30 May 2017 (registration number CRD42017058227), which was last updated on 6 July 2017 (for direct access to the final version and revision history, click on the following link http://www.crd.york.ac.uk/PROSPERO/display_record.asp?ID=CRD42017058227).

### Study eligibility

*Participants*: To be eligible, works must have examined type 2 diabetes patients with CKD.

*Intervention:* Intensive glycemic control.

*Comparators:* Standard glycemic control.

*Outcomes:* Primary outcome was doubling of serum creatinine. Secondary outcomes were need or start of dialysis and death from kidney failure.

*Study design:* RCTs and *post-hoc* analysis of RCTs comparing intensive vs. standard glycemic control were eligible works.

### Information sources and search strategy

PubMed, Ovid MEDLINE(R), Elsevier's Scopus, Web of Science, The Cochrane Central Register of Controlled Trials (CENTRAL), and ClinicalTrials.gov were searched from inception to May 2017.

Database-specific search strategies were developed using intervention search terms and the type of publication/document (click on the following link to view the complete search strategy https://www.crd.york.ac.uk/PROSPEROFILES/58227_STRATEGY_20170513.pdf).

Search strategies permitted also searches in DART-Europe E-Theses portal and Open Access Theses and Dissertations for identify relevant PhD and Masters Theses. Manual searches in the meeting abstracts archives of American Society of Nephrology—Kidney Week conferences 2003-2016, European Renal Association—European Dialysis and Transplantation Association (ERA-EDTA) congresses 2002-2016, and International Society of Nephrology (ISN) World Congresses of Nephrology 1999, 2001, 2003, 2005, 2007, 2009, 2011, 2013, 2015, and 2017, were conducted for other pertinent unpublished works.

Finally, to ensure literature saturation, a cited reference search of all eligible publications was carried out using Web of Science to identify all studies citing the included studies.

### Study selection and data collection

Screening of titles/abstracts and full text reports of potentially eligible articles in duplicate was carried out independently by FH-G and MA-G. Disagreements were resolved by discussion or referral to a third author (FJA). Corresponding authors of included studies were contacted whenever possible to retrieve missing information and to confirm study details.

### Data items and risk of bias assessment

An anonymized dataset describing study characteristics, sample characteristics, interventions, comparators, outcomes, and follow-up recorded during the trial was requested.

Assessment of risk of bias was made using the standard tool produced by the Cochrane Collaboration, including the domains of sequence generation, allocation concealment, blinding of participants or investigators, blinding of outcome assessors, completeness of outcome data, selective reporting, and other bias (Higgins et al., 2011).

### Data analysis

A systematic narrative synthesis was provided with information presented in the text and tables to summarize and explain the characteristics and findings of the included studies (AG-L, FH-G, MA-G). This narrative synthesis explores the relationship and findings both within and between the included studies, in line with the guidance from the Centre for Reviews and Dissemination at the University of York (2008).

Statistical analysis was performed by FH-G. Meta-analysis was carried out on aggregate data. Review Manager (RevMan) software version 5.3 (The Cochrane Collaboration) was used for analyses. The Mantel-Haenszel (M-H) random-effects model was used to estimate the pooled Odds Ratio (OR) with their 95% confidence interval (95% CI) for the following outcomes: (1) doubling of serum creatinine, (2) need or start of dialysis, and (3) death from kidney failure. Heterogeneity of the estimates was examined by computing the chi-squared statistic and quantifying inconsistency (I-squared statistic). Visual inspection of funnel plots of the estimates against their standard errors was made for assessing whether reporting bias was present.

## Results

**Figure 1:**
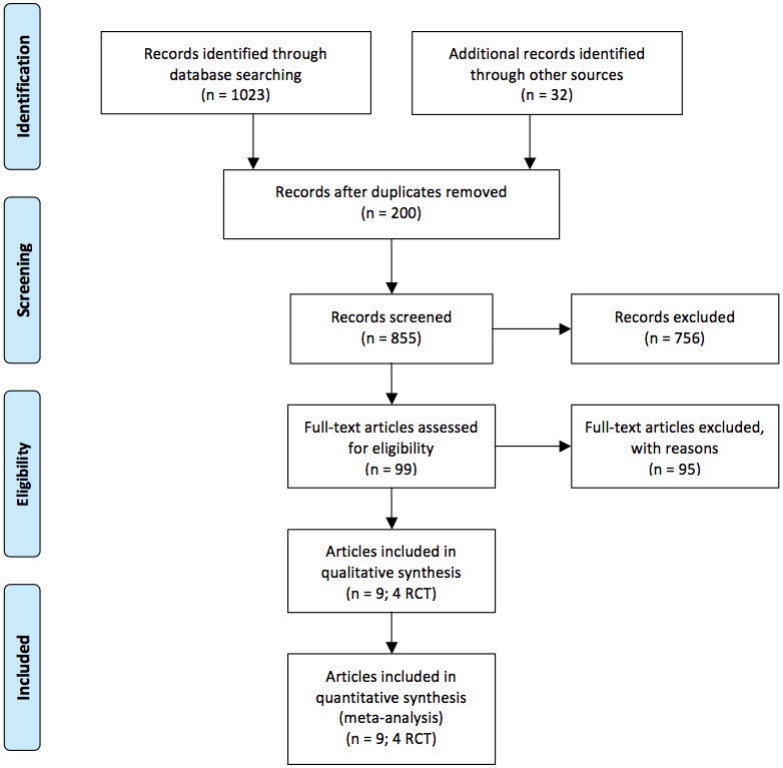
Flow chart of study selection process.

Our literature search found 855 unique citations. Of 99 potentially relevant full-text papers, 9 articles corresponding to four RCTs were selected (1998a; Adler et al., 2003; ADVANCE Collaborative Group, 2008; Holman et al., 2008; Duckworth et al., 2009; Ismail-Beigi et al., 2010; Perkovic et al., 2013; Papademetriou et al., 2015; Mohammedi et al., 2016). One PhD thesis contained data on one of these RCTs (Lambers Heerspink, 2008). Figure 1 presents the flow diagram of our search results according to PRISMA statement (Moher et al., 2009). All these studies were treat-to-target trials (Wangnoo et al., 2014). All were sponsored by state bodies involved in research from USA (Duckworth et al., 2009; Ismail-Beigi et al., 2010; Papademetriou et al., 2015), Australia (ADVANCE Collaborative Group, 2008; Perkovic et al., 2013; Mohammedi et al., 2016), and the United Kingdom (1998a; Adler et al., 2003; Holman et al., 2008), and were conducted at several collaborating centers across Asia, Australia, Europe, and North America.

### Characteristics of the participants

Table 1 summarizes and presents characteristics of eligible trials and of their participants, in addition to the interventions, comparators, outcomes, and follow-up.

**Table 1:** Participants, interventions, comparators, and outcomes in eligible trials.

**Trials**	**Design**	**Follow-up In years**	**Participants Characteristics (*n* or %)**	**Interventions (*n*)**	**Comparators (*n*)**	**Outcomes**	**Co-interventions**
**ACCORD**^#^ NCT00000620 (Ismail-Beigi et al., 2010; Papademetriou et al., 2015)	RCT (factorial design)	5 (3.5 in the intervention arm)	10-year mean duration T2DM patients (10,251). Age: 62.2 ± 6.8 years. NW^€^ (26.5%), previous CVE (35.2%), CKD/microalbuminuria (35.8/24.6%).	GLD^£^ (5,128)	Diet with/without GLD (5,123)	Glucose control. Blood pressure control. Lipid control.	Anti-hypertensive agents Fenofibrate or placebo plus simvastatin.
**ADVANCE**^‡^ NCT00145925 **ADVANCE-ON**^&^ NCT00949286 (ADVANCE Collaborative Group, 2008; Perkovic et al., 2013; Mohammedi et al., 2016)	RCT (factorial design) and PHA	5 (RCT) + 5.4 (PHA) = 9.9	8-year mean duration T2DM patients (11,140). Age: 66 ± 6 years. NW^€^ (31%), previous CVE (32.2%), CKD/microalbuminuria (44/26.8%).	GLD^£^ (5571)	Diet with/without GLD (5,569)	Glucose control. Blood pressure control.	Perindopril-indapamide.
**UKPDS**^§^ ISRCTN75451837 (1998a; Adler et al., 2003; Holman et al., 2008)	RCT and PHA	10 (RCT) + 10.7 (PHA) = 20.7	Newly diagnosed T2DM patients (4,209). Age: 53 ± 9 years. NW^€^ (19%), previous CVE (11%), CKD/microalbuminuria (11/6.5%).	GLD^*^ (3,071)	Diet without GLD if possible (1,549)	Glucose control.	–
**VADT**^$^ NCT00032487 (Duckworth et al., 2009)	RCT	5.6	11.5-year mean duration T2DM patients (1,791). Age: 60.5 ± 9 years. NW^€^ (38%), previous CVE (40%), CKD/microalbuminuria (41.5/19.7%).	GLD^£^ (899)	Diet with/without GLD (892)	Glucose control.	–

# *The Action to Control Cardiovascular Risk in Diabetes (ACCORD) trial*.

‡ *The Action in Diabetes and Vascular disease: PreterAx and DiamicroN Modified Release Controlled Evaluation (ADVANCE) trial*.

§ *The United Kingdom Prospective Diabetes Study (UKPDS)*.

$ *The Veterans Affairs Diabetes Trial (VADT)*.

€ *Enrollment of black or hispanic participants*.

£ *HbA_1c_ target-based titration*.

* *FPG target-based titration. CVE, cardiovascular event; FPG, fasting plasma glucose; GLD, glucose-lowering drugs; HbA_1c_, glycated hemoglobin; PHA, post-hoc analysis; RCT, randomized controlled trial; T2DM, type 2 diabetes mellitus*.

Twenty seven thousand three hundred and ninety one adult T2DM patients with or without CKD and modifiable cardiovascular risk factors or a previous cardiovascular event were randomly assigned to undergone either intensive glycemic control or standard glycemic control. Titrations of oral antidiabetic drugs (OAD) and/or insulin were left to the physician's discretion. The HbA_1c_ target levels of 6% (Duckworth et al., 2009; Ismail-Beigi et al., 2010; Papademetriou et al., 2015) or 6.5% (ADVANCE Collaborative Group, 2008; Perkovic et al., 2013; Mohammedi et al., 2016), and a fasting plasma glucose level less than 6 mmol/L (108 mg/dL) (1998a; Adler et al., 2003; Holman et al., 2008) defined intensive control of glycemia. Treatment protocols advised the use of a sulfonylurea (metformin in overweight and obese patients) with the increase in doses or the addition of any other OAD, or insulin injections if the glycemic control was not achieved (1998a; Adler et al., 2003; ADVANCE Collaborative Group, 2008; Holman et al., 2008; Duckworth et al., 2009; Ismail-Beigi et al., 2010; Perkovic et al., 2013; Papademetriou et al., 2015; Mohammedi et al., 2016).

At randomization, according to the definition and classification system for CKD provided by the Kidney Disease: Improving Global Outcomes (KDIGO) CKD Work Group in their clinical practice guidelines (Kidney Disease, 2013), participants had an estimated glomerular filtration rate (eGFR) of 90 mL/min per 1.73 m^2^ or more with an albumin—creatinine ratio (ACR) of 29 μg/mg or less (CKD stage 1) (1998a; Adler et al., 2003; Holman et al., 2008), an eGFR of 60–89 mL/min per 1.73 m^2^, and an ACR ≥ 30 μg/mg (CKD stage 2) (1998a; Adler et al., 2003; ADVANCE Collaborative Group, 2008; Holman et al., 2008; Duckworth et al., 2009; Ismail-Beigi et al., 2010; Perkovic et al., 2013; Papademetriou et al., 2015; Mohammedi et al., 2016), or an eGFR of 30–59 mL/min per 1.73 m^2^ with or without albuminuria (CKD stage 3) (Ismail-Beigi et al., 2010; Papademetriou et al., 2015). Microalbuminuria was present at baseline, mostly among patients being diabetic for 8–10 years or more (ADVANCE Collaborative Group, 2008; Duckworth et al., 2009; Ismail-Beigi et al., 2010; Perkovic et al., 2013; Papademetriou et al., 2015; Mohammedi et al., 2016), and among those having poor glycemic control (Duckworth et al., 2009). Probability of progression from normoalbuminuria to microalbuminuria was most important compared to directly transition from no nephropathy to macroalbuminuria (Adler et al., 2003).

The trials eligible are individually less convincing to attribute an impact of glucose-lowering drugs on eGFR compared to the effect on albuminuria, and this was particularly important among the Veterans Affairs Diabetes Trial (VADT) participants (Duckworth et al., 2009).

Follow-up times varied substantially across trials. The intensive glycemic control arm of the Action to Control Cardiovascular Risk in Diabetes (ACCORD) trial was stopped 17 months before the scheduled end of the study because of the increase in all-cause mortality, and patients were transitioned to standard therapy until the planned end of 5 years (Action to Control Cardiovascular Risk in Diabetes Study Group, 2008). All surviving patients from the total who were included in both The Action in Diabetes and Vascular disease: PreterAx and DiamicroN Modified Release Controlled Evaluation (ADVANCE) trial (ADVANCE Collaborative Group, 2008; Perkovic et al., 2013) and The United Kingdom Prospective Diabetes Study (UKPDS) (1991; 1998a; 1998b; 1999; Adler et al., 2003) entered in post-trial monitoring studies, and had a complete follow-up, respectively, of 9.9 years (Mohammedi et al., 2016) and 20 years (Holman et al., 2008).

### Assessment of risk of bias

Results for each domain and each trial are given in Table 2. Only the ADVANCE trial adopted a double-blind design (ADVANCE Collaborative Group, 2008; Perkovic et al., 2013; Mohammedi et al., 2016), but risk of bias from lack of blinding of participants and personnel in the remaining trials was judged as unclear considering the type of outcomes assessed. All the included trials failed also to report blinding of outcome assessors. Finally, all trials were funded by companies that produce glucose-lowering medications, and were considered as having unclear risk of bias for the other sources of bias domain.

**Table 2:** Appraising the risk of bias in eligible trials using the Cochrane risk of bias tool.

**Trials**	**Random sequence generation**	**Allocation concealment**	**Blinding of participants ans personnel**	**Blinding of outcome assessment**	**Incomplete outcome data**	**Selective reporting**	**Other bias**
**ACCORD**	Low risk of bias	Low risk of bias	Unclear risk of bias	Unclear risk of bias	Low risk of bias	Low risk of bias	Unclear risk of bias
**ADVANCE**	Low risk of bias	Low risk of bias	Low risk of bias	Unclear risk of bias	Low risk of bias	Low risk of bias	Unclear risk of bias
**UKPDS**	Low risk of bias	Low risk of bias	Unclear risk of bias	Unclear risk of bias	Low risk of bias	Low risk of bias	Unclear risk of bias
**VADT**	Low risk of bias	Low risk of bias	Unclear risk of bias	Unclear risk of bias	Low risk of bias	Low risk of bias	Unclear risk of bias

### Clinical kidney disease outcomes

**Figure 2:**
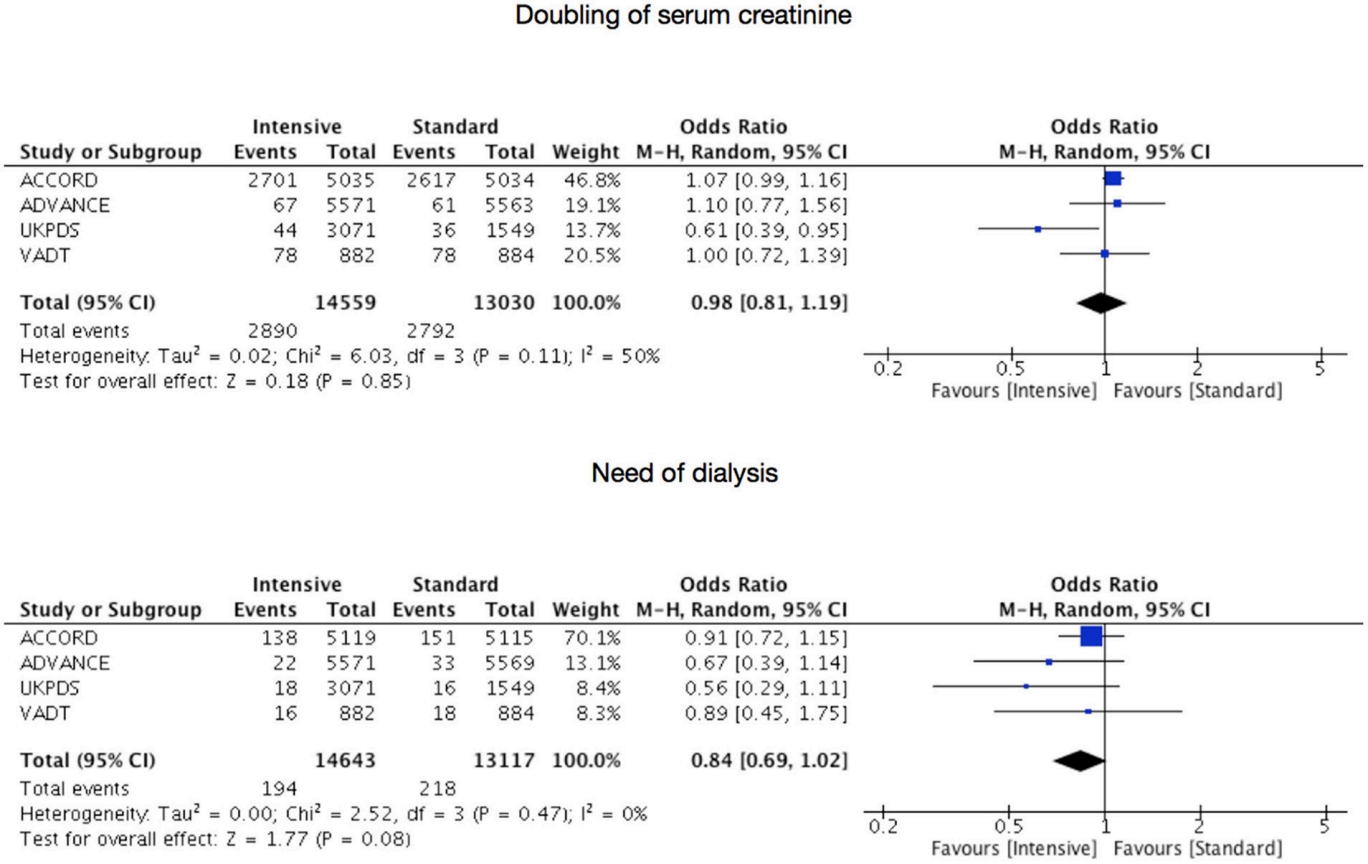
Risks for doubling of serum creatinine or start of dialysis under intensive glycemic control.

**Figure 3:**
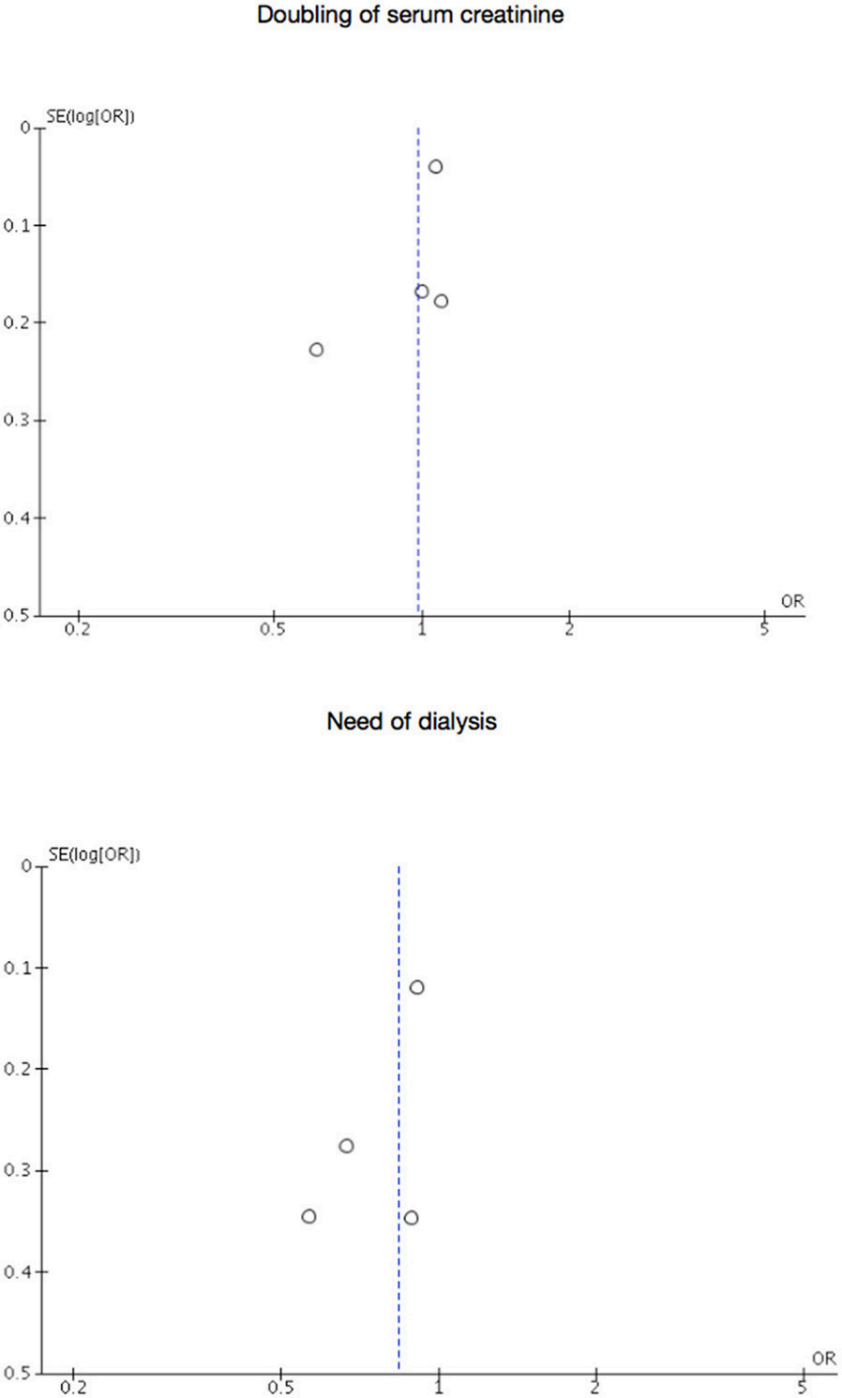
Funnel plots for doubling of serum creatinine or start of dialysis.

All RCTs assessed the outcomes of doubling of serum creatinine and need of dialysis. Figures 2, 3 present, respectively, forest plots and funnel plots for these outcomes.

Serum creatinine doubled in 2,890 patients of a total of 14,559 in the intensive therapy group (19.85%) vs. 2,792 of a total of 13,030 (21.43%) in the standard therapy group, being the pooled OR of 0.98 with 95% CI 0.81–1.19 (*p* = 0.32, *I*^2^ = 15%). 194 of the 14,643 in the intensive therapy group (1.32%) started dialysis compared to 218 of the 13,117 (1.66%) in the standard therapy group, being the pooled OR of 0.84 with 95% CI 0.69–1.02 (*p* = 0.47, *I*^2^ = 0%). Asymmetrical funnel plots place most included trials toward the top of diagrams (larger, most powerful studies).

**Figure 4:**
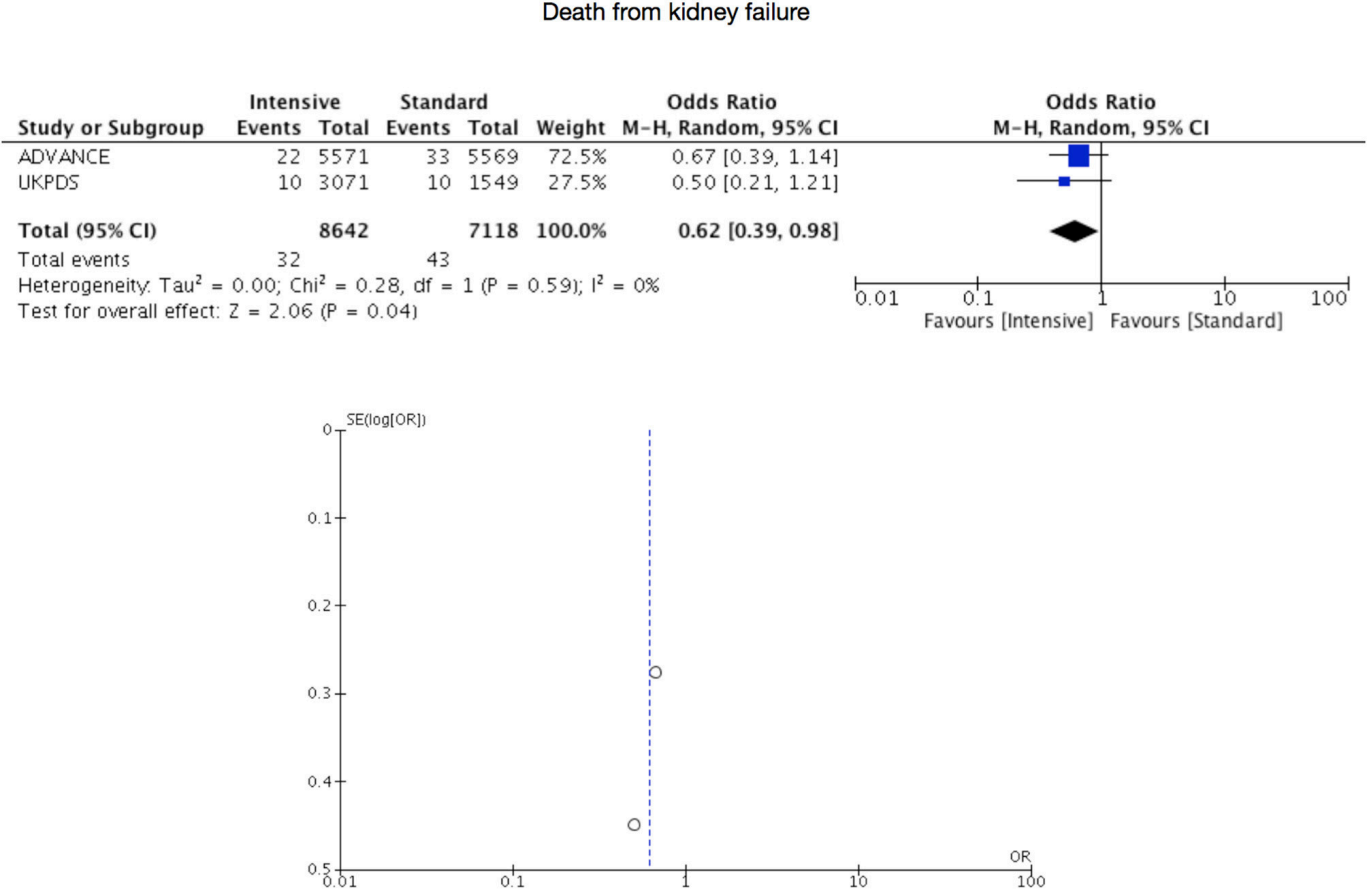
Risk for death from kidney failure.

Only two RCTs provided numerical data for the outcome of death from kidney failure (1998a; Adler et al., 2003; ADVANCE Collaborative Group, 2008; Holman et al., 2008; Perkovic et al., 2013; Mohammedi et al., 2016). Thirty-two events (*n* = 8,642) in the intensive therapy group and 43 events (*n* = 7,118) in the standard therapy group were recorded, being the pooled OR of 0.62 with 95% CI 0.39–0.98 (p = 0.04, *I*^2^ = 0%, Figure 4).

## Discussion

Our results provide comprehensive and detailed evidence on the differential effect of intensive and standard glycemic control on CKD of T2DM patients. There was a favorable impact of intensive therapy on death from kidney failure. Nevertheless, the proportions of patients from the included RCTs under intensive therapy that doubled serum creatinine or needed dialysis were comparable to the number of patients under standard therapy in whom these outcomes occurred. These large trials were also individually less convincing to attribute a favorable impact of pharmacological treatment on the clinically manifest CKD (1998a; Adler et al., 2003; ADVANCE Collaborative Group, 2008; Holman et al., 2008; Duckworth et al., 2009; Ismail-Beigi et al., 2010; Perkovic et al., 2013; Papademetriou et al., 2015; Mohammedi et al., 2016), while an effect on albuminuria could be observed, or was better appreciated in selected populations (longer duration of diabetes, poor glycemic control, etc.) (Duckworth et al., 2009).

Gylcemic control has effects on microvascular complications of diabetes (Klein et al., 1984; Ballard et al., 1988; Diabetes Control and Complications Trial Research Group, 1993; Ohkubo et al., 1995; Adler et al., 1997). However, until now only effects on albuminuria had presented (Diabetes Control and Complications Trial Research Group, 1993; Ohkubo et al., 1995; Duckworth et al., 2009). While based on only two RCTs, our work show that an effect on death is also evident, considering that other factors may influence on the course of this disease. Probably, transition across CKD stages to End-Stage Kidney Disease (ESKD) occur independently of control of glycemia once there is a structural damage of the kidney. This may be the reason for the inconclusive values of pooled OR for the outcomes of doubling of serum creatinine and need of dialysis. Nevertheless, intensive regimens may have a detrimental impact in clinical CKD (Action to Control Cardiovascular Risk in Diabetes Study Group, 2008; ADVANCE Collaborative Group, 2008; Duckworth et al., 2009). Risk for hypoglycemia that increases as CKD progresses, may explain why limits should be established for more intensive control strategies (National Kidney Foundation, 2012). In any case, an optimal glycemic control is a hard task, particularly if diet is not sufficient and any dose of insulin is not tolerated (Rhee et al., 2014).

Importantly, glucose-lowering medications assessed in the included trials were different to newer anti-diabetic drugs, especially concerning safety. For instance, compared to sulfonylureas and meglitinides, dipeptidyl peptidase-4 (DPP-4) inhibitors provide important reduction in HbA_1c_ concentration, with a low risk for hypoglycemia, even in advanced CKD patients (eGFR < 30 mL/min per 1.73 m^2^) (Russo et al., 2013; Scheen, 2015). However, most DPP-4 inhibitors (sitagliptin, vildagliptin, saxagliptin, alogliptin) are predominantly excreted by the kidneys, and dose reductions are needed according to the severity of CKD (Scheen, 2015). Larger clinical trials are ongoing to confirm potential benefits of these medications, although it seems that only improvement in albuminuria is associated with it (Penno et al., 2016). A mention should also be made for glucagon-like peptide-1 (GLP-1) receptor agonists, which must be used with caution, especially in aged diabetic patients, because of their gastrointestinal adverse effects (Panduru et al., 2017). However, these drugs seem to improve cardiovascular disease and their once-weekly use is quite attractive. Empagliflozin, a sodium–glucose co-transporter 2 (SGLT-2) inhibitor, have also the advantage of low risk for hypoglycemia, but their efficacy is reduced in CKD because this depends among other things on hypovolemia that may be associated with kidney function deterioration (Panduru et al., 2017). In any case, insulinization remains the last and the most appropriate option for an adequate glycemic control in advanced CKD, if there is not risk for hypoglycemia conforming to current guidelines (National Kidney Foundation, 2012).

Titration of medications in these treat-to-target trials is an important feature allowing the evaluation of the risk of complications between these treatment modalities oriented toward achieving a well-defined physiologic target (Wangnoo et al., 2014). Different titration algorithms have been used in clinical trials in diabetes (Strange, 2007), but physician-directed titration seems to be more plausible in the context of clinical practice where the identification of prognostic outcomes such as progression of CKD proves to be important. In this sense, the fact of have a normal kidney function provides an advantage (Action to Control Cardiovascular Risk in Diabetes Study Group, 2008; ADVANCE Collaborative Group, 2008; Duckworth et al., 2009; Jun et al., 2011; Alsahli and Gerich, 2014). However, diabetes is the leading cause of CKD and poor control of glycemia is associated with a more rapid entrance in CKD. Therefore, the knowledge of the impact of glycemic control in both populations with and without CKD should be considered as an important aspect, as it might be interesting for health technology assessment (HTA) actors involved in the evaluation of such therapies.

This meta-analysis has been carried out according to a planned, registered and prospectively updated review protocol (Shamseer et al., 2015), as a clear signal of maintaining transparency in the systematic review process (Stewart et al., 2012), avoiding future changes which may be associated with reporting biases (Kirkham et al., 2010), and showing the suitability and non-duplicity of our analysis (Moher, 2013; Siontis et al., 2013). In addition, eligible trials had a good quality, so it is possible to rely on the summary of these.

Nevertheless, two biased and unregistered systematic reviews present assessments of some of the outcomes considered in this meta-analysis (Coca et al., 2012; Slinin et al., 2012). One analysis failed to include all relevant studies (Slinin et al., 2012), and the other one duplicated the data from an included study (Coca et al., 2012). In addition, a corrected version of one of these works did not contribute to correct errors, because it was only centered in some aspects of study eligibility (Coca et al., 2012). Taking account of unreliability of pooled estimates from these studies and contrarily to methods followed in them and their conduct, data for our analysis was obtained using a highly sensitive, transparent, and reproducible literature search strategy, which is in addition publicly available. Searches in gray literature sources guarantee that potentially all studies answering the review question have been retrieved. Nevertheless, reporting bias is more likely to be the cause of asymmetrical funnel plots observed, although in diagrams there is a suggestion of missing studies on areas of higher significance, which explain that asymmetry is probably due to factors other than reporting bias (Sterne et al., 2011). Publication bias and related biases can lead to overly optimistic conclusions in systematic reviews, but visual inspection of funnel plots is not usually made correctly (Terrin et al., 2005), and not interpreted considering the circumstances in which the intervention was implemented in the included studies or in the context of susceptibility to biases (Sterne et al., 2011). Heterogeneity may also lead to funnel plot asymmetry, but differences both in patients' characteristics and in the interventions were not translated in statistical heterogeneity and permit generalizability of the findings of our meta-analysis (Higgins et al., 2003). Finally, it must not be forgotten that only four RCTs were eligible, and this fact might lead to include observational evidence being of low quality (O'Neil et al., 2014).

## Conclusions

Intensive glycemic control has an effect on death from kidney failure compared to standard glycemic control. However, there is apparently no effect on the outcomes of doubling of serum creatinine and need of dialysis. Probably, other factors that influence on the course of CKD in T2DM patients, may provide the explanation of this absence of effect. CKD itself seems to be the most important impediment for an adequate glycemic control in diabetics. Our observations stress the need for a better comprehension of glycemic control effects on both T2DM patients with and without CKD for individualization of these two treatment modalities. From the perspective of guidelines, it is legitimate to recommend managing glycemia cautiously in CKD, especially in patients with eGFR < 30 mL/min per 1.73 m^2^, as progression to ESKD occurs independently of glycemic control, and particularly because there is an increasing risk for hypoglycemia as CKD progresses.

## Author contributions

FH-G developed the hypothesis and study design. FH-G, FJA, and MA-G performed the literature searches and/or screened papers. AG-L, FH-G, and MA-G realized data acquisition and synthesis. FH-G performed statistical analyses. FH-G and MA-G drafted the manuscript. All authors have given final approval for this paper to be published.

### Conflict of interest statement

The authors declare that the research was conducted in the absence of any commercial or financial relationships that could be construed as a potential conflict of interest.
